# Multidisciplinary management of difficult-to-treat drug resistant tuberculosis: a review of cases presented to the national consilium in Uganda

**DOI:** 10.1186/s12890-021-01597-1

**Published:** 2021-07-10

**Authors:** Joseph Baruch Baluku, Richard Katuramu, Joshua Naloka, Enock Kizito, Martin Nabwana, Felix Bongomin

**Affiliations:** 1 Division of Pulmonology, Kiruddu National Referral Hospital, Kampala, Uganda; 2 Makerere University Lung Institute, Kampala, Uganda; 3 Uganda Ministry of Health, Kampala, Uganda; 4 Mulago National Referral Hospital, Kampala, Uganda; 5 USAID/Defeat TB, University Research Co LLC, Kampala, Uganda; 6 Makerere University – Johns Hopkins University Research Collaboration, Kampala, Uganda; 7 Department of Medical Microbiology and Immunology, Faculty of Medicine, Gulu University, Gulu, Uganda

**Keywords:** Consilium, Difficult to treat, Multidisciplinary teams, MDR TB, Uganda, Outcomes

## Abstract

**Background:**

Patients with drug resistant tuberculosis (DR-TB) with comorbidities and drug toxicities are difficult to treat. Guidelines recommend such patients to be managed in consultation with a multidisciplinary team of experts (the “TB consilium”) to optimise treatment regimens. We describe characteristics and treatment outcomes of DR-TB cases presented to the national DR-TB consilium in Uganda between 2013 and 2019.

**Methods:**

We performed a secondary analysis of data from a nation-wide retrospective cohort of DR-TB patients with poor prognostic indicators in Uganda. Patients had a treatment outcome documented between 2013 and 2019. Characteristics and treatment outcomes were compared between cases reviewed by the consilium with those that were not reviewed.

**Results:**

Of 1,122 DR-TB cases, 189 (16.8%) cases from 16 treatment sites were reviewed by the consilium, of whom 86 (45.5%) were reviewed more than once. The most frequent inquiries (N = 308) from DR-TB treatment sites were construction of a treatment regimen (38.6%) and management of side effects (24.0%) while the most frequent consilium recommendations (N = 408) were a DR-TB regimen (21.7%) and “observation while on current regimen” (16.6%). Among the cases reviewed, 152 (80.4%) were from facilities other than the national referral hospital, 113 (61.1%) were aged ≥ 35 years, 72 (40.9%) were unemployed, and 26 (31.0%) had defaulted antiretroviral therapy. Additionally, 141 (90.4%) had hepatic injury, 55 (91.7%) had bilateral hearing loss, 20 (4.8%) had psychiatric symptoms and 14 (17.7%) had abnormal baseline systolic blood pressure. Resistance to second-line drugs (SLDs) was observed among 9 (4.8%) cases while 13 (6.9%) cases had previous exposure to SLDs. Bedaquiline (13.2%, n = 25), clofazimine (28.6%, n = 54), high-dose isoniazid (22.8%, n = 43) and linezolid (6.7%, n = 13) were more frequently prescribed among cases reviewed by the consilium than those not reviewed. Treatment success was observed among 126 (66.7%) cases reviewed.

**Conclusion:**

Cases reviewed by the consilium had several comorbidities, drug toxicities and a low treatment success rate. Consilia are important “gatekeepers” for new and repurposed drugs. There is need to build capacity of lower health facilities to construct DR-TB regimens and manage adverse effects.

## Background

Drug resistant tuberculosis (DR-TB) is a threat to tuberculosis (TB) control with ~ 500,000 cases of rifampicin resistance (RR) reported in 2019 [1]. In some parts of sub-Saharan Africa (SSA), the prevalence of DR-TB has been reported to be 20% among new TB patients, 53% among previously treated TB patients and the rate of decline of multi-drug resistant TB (MDR-TB) in SSA is dismal [2, 3]. The MDR-TB treatment success rate is low and only 57% of patients on conventional treatment regimens have a successful outcome [4].

Comorbid conditions such as human immunodeficiency virus (HIV) infection, diabetes mellitus, cancer, alcohol and cigarette use, in addition to cavitary TB disease, resistance to second-line drugs (SLDs), pre- and extensively drug resistant TB (XDR-TB) and drug adverse effects complicate management of DR-TB and contribute to the poor outcomes [5–10]. Primary health workers in low-income settings find patients with these conditions to be difficult-to-treat and expert opinion is often needed. Such patients may require substitution of drugs in the standard regimen, dose adjustment, discontinuation of a single agent or inclusion of less efficacious agents in the treatment regimen [10–12].

A survey of TB and MDR/XDR-TB management in European countries revealed that there was significant discordance between guidelines and the practice of health care workers in regimen selection (characterised by fewer than 4 drugs in the regimen, incorrect drug choice, inadequate dose prescription, short treatment duration and inadequate management of adverse events of treatment), anti-retroviral therapy (ART) prescription in the HIV co-infected and documentation of treatment outcomes [13]. Similar discordance between guidelines and practice in DR-TB management has been reported in SSA [14]. The World Health Organization (WHO) recommends the formation of national clinical review committees (TB consilia) consisting of multidisciplinary teams that include representation from health care professionals that have expertise in surgery, paediatrics, adult medicine, radiology, psychology, nutrition, and nursing among others [15]. The clinical review committee is a formal system of consultation and provides consensus on evidence-based treatment approaches for difficult-to-treat patients [16]. With the advent of new anti-TB drugs, the consilia are thought to be key as “gate keepers” to guide the judicious use of these agents to protect them from resistance [17]. Primary health care workers can access the input of the consilia through a phone, post office, email, web or physical meetings [17].

In Uganda, such a consilium (also known as a DR-TB review panel) is expected to hold monthly in-person meetings to discuss complex cases referred from DR-TB treatment sites across the country [18]. Despite the utility of these consilia, there has been no nationwide evaluation of their impact on DR-TB treatment outcomes of difficult-to-treat patients in low-income settings. The reports available [16, 19] are mostly from high-income countries with low TB/HIV burden, yet formulation and functionality of consilia in resource-limited settings pose time and human resource constraints in an already overstretched health system.

In this study, we describe characteristics and treatment outcomes of DR-TB cases presented to the national DR-TB consilium in Uganda drawn from a large nationwide cohort [20] of patients with poor prognostic indicators, of whom 59% were HIV co-infected.

## Methods

### Study population and design

We conducted secondary analysis of data from a nationwide retrospective cohort [20] of DR-TB patients with poor prognostic indicators who had laboratory confirmed DR-TB and a treatment outcome documented between January 2013 and December 2019. We extracted data for patients reviewed by the national DR-TB consilium during the same period. Because we sought to compare characteristics of cases reviewed with those that were not reviewed, no cases were excluded in the current analysis.

### The DR-TB consilium in Uganda

The national DR-TB consilium in Uganda is hosted at Mulago National Referral Hospital (MNRH) tuberculosis unit. MNRH is in Kampala, the capital city of Uganda and the TB unit serves as the centre of excellence for susceptible TB and DR-TB care to which all other units of the hospital and peripheral facilities refer patients for diagnosis and TB treatment. There are 16 other DR-TB treatment centres in the country that comprise of 13 rural regional referral hospitals and three general district hospitals. At the time of conducting the study, the national DR-TB consilium in Uganda comprised of two pulmonologists, two internal medicine physicians, a paediatric TB specialist, two audiologists, a laboratory technologist from the national tuberculosis reference laboratory, social worker, the national DR-TB coordinator, a radiology resident, three TB community linkage facilitators, adult and paediatric TB nurses and the National TB and Leprosy Program manager. The consilium held in-person meetings every 2 weeks to discuss difficult-to-treat cases. The difficult-to-treat cases were referred from all DR-TB treatment centres (including MNRH). All DR-TB treatment centers in Uganda were recommended to have local expert panels to review all patients initiating DR-TB treatment but the composition of local panels depended on the expertise available at each center.

Case summaries were sent by email to the coordinator of the meetings of the national consilium from the DR-TB treatment centers. These were presented to the consilium by a medical officer. The case summary typically had the patient’s medical history, most relevant clinical examination findings, and laboratory and imaging investigations done. The summary highlighted the clinical inquiry for which the treatment site is consulting about. After deliberation, an action plan was drawn and was communicated by phone or e-mail by the meeting secretary to the referring treatment site which documented the consilium recommendations in the patient chart and executed the proposed management plan. In January 2019, the consilium started using video tele-conferencing (Project ECHO (University of New Mexico)) with the DR-TB treatment sites in which the individual site representative presented the case summary of the difficult-to-treat case to a physical meeting of the consilium members. The consilium has been in existence since 2013.

### Study measurements

In the primary study, data were abstracted from participant charts using a data abstraction form. In the current analysis, demographic data (level of DR-TB treatment facility, age, sex, employment status, and marital status), clinical characteristics (alcohol and cigarette use, HIV status, HIV treatment details, TB resistance profiles, DR-TB treatment details, liver function tests, haemoglobin levels, and  audiograms), treatment outcomes (cured, treatment completion, loss-to-follow-up, and death) and consilium review details were abstracted. For the consilium review, the number of times the case was reviewed was determined. The total number of inquiries and recommendations of the panel were also enumerated. Full details of the primary study measurements and programmatic management of DR-TB in Uganda are provided elsewhere [20, 21]. Treatment outcomes were defined according to WHO definitions [22].

### Statistical analysis

Data were analysed with Stata 15.0 (STATA, College Station, Texas, USA). We used descriptive statistics and reported frequencies and proportions alongside the 95% confidence intervals (CI). Characteristics and outcomes were compared between cases reviewed by the consilium and those not reviewed.

## Results

Data on 1,112 DR-TB cases were extracted from the primary data set. Of these, 189 (16.8%) were reviewed by the national DR-TB consilium while 923 (83.2%) were not reviewed.

### Characteristics of patients reviewed by the national DR-TB consilium in Uganda

Among those reviewed, 86 (45.5%) were reviewed more than once, 172 (91.0%) had RR/MDR-TB, 10 (5.3%) had pre-XDR-TB, 3 (1.6%) had XDR-TB, 4 (2.1%) had poly-resistant TB and 102 (54.0%) were HIV co-infected. More characteristics are shown in Tables 1 and 2.

**Table 1:** A comparison of sociodemographic characteristics of DR-TB cases reviewed by the national DR-TB consilium with those not reviewed

Socio-demographic characteristic	Review status			
	Not reviewed n = 933	95% CI	Reviewed n = 189	95% CI
*Level of treatment site*				
Mulago National Referral Hospital	284 (30.4)	27.6, 33.5	37 (19.6)	14.5, 25.9
Regional referral hospital	533 (57.1)	53.9, 60.3	120 (63.5)	56.3, 70.1
District hospital	116 (12.4)	10.5, 14.7	32 (16.9)	12.2, 23.0
Residence, *n* = *1076*				
Rural	583 (65.1)	62.0, 68.2	113 (62.4)	55.1, 69.2
Urban	312 (34.9)	31.8, 38.0	68 (37.6)	30.8, 44.9
Age, *n* = *1080*				
< 15	22 (2.5)	1.6, 3.7	5 (2.7)	1.1, 6.4
15–34	387 (43.2)	40.0, 46.5	67 (36.2)	29.6, 43.4
35–60	449 (50.2)	46.9, 53.4	94 (50.8)	43.6, 58.0
> 60	37 (4.1)	3.0, 5.7	19 (10.3)	6.6, 15.6
*Sex*				
Male	590 (63.2)	60.1, 66.3	119 (63)	55.8, 69.6
Female	343 (36.8)	33.7, 39.9	70 (37)	30.4, 44.2
Pregnancy status*, n = 413				
Pregnant	13 (3.8)	2.2, 6.4	5 (7.1)	3.0, 16.3
Breastfeeding	11 (3.2)	1.8, 5.7	5 (7.1)	3.0, 16.3
None	319 (93)	89.8, 95.3	60 (85.7)	75.2, 92.2
*Nature of employment, n = 1060*				
Unemployed	280 (31.7)	28.7, 34.8	72 (40.9)	33.8, 48.4
Self employed	402 (45.5)	42.2, 48.8	73 (41.5)	34.4, 48.9
Employed	202 (22.9)	20.2, 25.7	31 (17.6)	12.6, 24.0
*Marital Status, n = 1069*				
Married	446 (50.6)	47.3, 53.9	89 (47.3)	40.3, 54.5
Single	217 (24.6)	21.9, 27.6	50 (26.6)	20.7, 33.4
Divorced	25 (2.8)	1.9, 4.2	3 (1.6)	0.5, 4.9
Separated	137 (15.6)	13.3, 18.1	39 (20.7)	15.5, 27.2
Widowed	56 (6.4)	4.9, 8.2	7 (3.7)	1.8, 7.6
*Alcohol use, n = 816*				
Never	412 (62)	58.2, 65.6	91 (60.3)	52.2, 67.8
Ever used	253 (38)	34.4, 41.8	60 (39.7)	32.2, 47.8
*Cigarette smoking, n = 816*				
Never	541 (81.4)	78.2, 84.1	120 (79.5)	72.2, 85.2
Ever used	124 (18.6)	15.9, 21.8	31 (20.5)	14.8, 27.8

^*^Among females, CI–confidence interval

**Table 2:** A comparison of clinical characteristics the DR-TB cases reviewed by the national DR-TB consilium with those not reviewed

Clinical characteristic	Review status			
	Not reviewed n = 933	95% CI	Reviewed n = 189	95% CI
*Body mass index, kg/m*^*2*^* n = 473*				
< 18.5	195 (55.9)	50.6, 61.0	81 (65.3)	56.5, 73.2
18.5–24.9	142 (40.7)	35.6, 45.9	39 (31.5)	23.8, 40.2
25.0–29.9	12 (3.4)	2.0, 6.0	4 (3.2)	1.2, 8.4
*History of TB treatment*				
Yes	556 (59.6)	56.4, 62.7	107 (56.6)	49.4, 63.5
No	377 (40.4)	37.3, 43.6	82 (43.4)	36.5, 50.6
HIV Status				
Positive	564 (60.5)	57.3, 63.5	102 (54)	46.8, 61.0
Negative	369 (39.5)	36.5, 42.7	87 (46)	39.0, 53.2
*Cotrimoxazole use, n = 603*				
Yes	491 (97)	95.1, 98.2	96 (99)	92.9, 99.9
No	15 (3)	1.8, 4.9	1 (1)	0.1, 7.1
*ART use, n = 655*				
Yes	521 (94)	91.7, 95.7	101 (100)	-
No	33 (6)	4.3, 8.3	0 (0)	-
*History of ART default, n = 491*				
Yes	60 (14.7)	11.6, 18.5	26 (31)	21.9, 41.8
No	347 (85.3)	81.5, 88.4	58 (69)	58.2, 78.1
Diabetes, *n* = *151*				
Yes	36 (33)	24.8, 42.5	12 (28.6)	16.7, 44.4
No	73 (67)	57.5, 75.2	30 (71.4)	55.6, 83.3
*Hypertension, n = 479*				
Yes	19 (4.6)	2.9, 7.0	5 (8.1)	3.3, 18.2
No	398 (95.4)	93.0, 97.1	57 (91.9)	81.8, 96.7
*Systolic blood pressure, n = 479*				
< 90	32 (7.7)	5.5, 10.7	10 (16.1)	8.8, 28.7
90–140	375 (89.9)	86.6, 92.5	48 (77.4)	65.1, 86.3
> 140	10 (2.4)	1.3, 4.4	4 (6.5)	2.4, 16.2
*Diastolic blood pressure, n = 479*				
< 60	41 (9.8)	7.3, 13.1	10 (16.1)	8.8, 27.7
60–90	362 (86.8)	83.2, 89.7	51 (82.3)	70.5, 90.0
> 90	14 (3.4)	2.0, 5.6	1 (1.6)	0.2, 11.0
*Cancer*				
No	919 (98.5)	97.5, 99.1	186 (98.4)	95.2, 99.5
Yes	14 (1.5)	0.9, 2.5	3 (1.6)	0.5, 4.8
*Elevated creatinine*^*†*^*, n = 934*				
Yes	231 (29.4)	26.3, 32.6	48 (32.7)	25.5, 40.7
No	556 (70.6)	67.4, 73.7	99 (67.3)	59.3, 74.5
*Hepatic injury*^*§*^*, n = 928*				
Yes	639 (82.8)	79.9, 85.3	141 (90.4)	84.6, 94.1
No	133 (17.2)	14.7, 20.1	15 (9.6)	5.9, 15.4
*Hearing impairment, n = 803*				
Normal Hearing	351 (54.5)	50.6, 58.3	93 (58.5)	50.6, 65.9
Low Frequency	113 (17.5)	14.8, 20.7	24 (15.1)	10.3, 21.6
High Frequency	180 (28)	24.6, 31.6	42 (26.4)	20.1, 33.9
*Distribution of hearing loss, n = 348*				
Bilateral hearing loss	233 (80.9)	75.9, 85.1	55 (91.7)	81.2, 96.6
Unilateral hearing loss	55 (19.1)	14.9, 24.1	5 (8.3)	3.4, 18.8
*Grade of hearing loss, n = 314*				
Mild	145 (55.3)	49.2, 61.3	20 (38.5)	26.1, 52.6
Moderate	65 (24.8)	19.9, 30.4	18 (34.6)	22.8, 48.7
Severe	34 (13)	9.4, 17.6	9 (17.3)	9.1, 30.4
Profound	18 (6.9)	4.4, 10.7	5 (9.6)	4.0, 21.5
*Psychiatric symptoms*				
Yes	49 (5.3)	4.0, 6.9	20 (10.6)	6.9, 15.9
No	884 (94.7)	93.1, 96.0	169 (89.4)	84.1, 93.1
*Resistance to SLDs*				
Yes	4 (0.4)	0.1, 1.1	9 (4.8)	2.5, 8.9
No	929 (99.6)	98.9, 99.8	180 (95.2)	91.1, 97.5
*Previous exposure to SLDS*				
Yes	17 (1.8)	1.1, 2.9	13 (6.9)	4.0, 11.5
No	916 (98.2)	97.1, 98.9	176 (93.1)	88.5, 96.0

SLDs–second-line drugs,TB–tuberculosis, DR-TB–drug resistant TB, ART–antiretroviral therapy, SLDs–second-line drugs, ^†^defined as creatinine level of > 106.1 µmol per litre (µm/l) [23], ^§^ defined as elevation in any of total bilirubin, aspartate aminotransferase, gamma-glutamyl transferase, alanine aminotransferase and alkaline phosphatase at the respective cut offs of: > 2.5 mg/dl, > 34.8 units per litre (U/L), > 68.5 U/L, > 42.8 U/L and > 151 U/L [23]

### Comparison of consilium-reviewed and non-reviewed cases

As shown in Tables 1 and 2, among the cases reviewed, 152 (80.4%) were from facilities other than the national referral hospital, 113 (62.4%) were from rural areas, 113 (61.1%) were aged ≥ 35 years, 72 (40.9%) were unemployed, and 26 (31.0%) had defaulted antiretroviral therapy. Additionally, 141 (90.4%) had hepatic injury, 55 (91.7%) had bilateral hearing loss, 20 (4.8%) had psychiatric symptoms and 14 (17.7%) had abnormal systolic blood pressure. Resistance to second-line drugs (SLDs) was observed among 9 (4.8%) cases while 13(6.9%) cases had previous exposure to SLDs. Bedaquiline (13.2%, n = 25), clofazimine (28.6%, n = 54), high-dose isoniazid (22.8%, n = 43) and linezolid (6.7%, n = 13) were more frequently prescribed among cases reviewed by the consilium than those not reviewed.

### Treatment outcomes of cases reviewed by the national DR-TB consilium

Treatment success was observed among 126 (66.7%) cases reviewed by the consilium. In comparison, treatment success was observed among 680 (72.9%) cases that were not reviewed by the consilium. Treatment loss-to-follow-up was observed among 18 (9.5%) cases reviewed while treatment failure and death occurred among 9 (4.8%) and 36 (19.0%) cases reviewed respectively.

### Clinical inquiries from DR-TB sites and recommendations of the consilium

The most frequent inquiries (N = 308) from DR-TB sites were construction of a treatment regimen (38.6%), management of side effects (24.0%) and determination of treatment duration (21.8%). The most frequent recommendations (N = 408) were a DR-TB regimen (21.7%), observation while on current regimen (16.6%) and treatment adherence counselling (14.0%). Notably, the number of inquiries and recommendations were more than the number of cases reviewed because some cases were reviewed more than once and had more than one inquiry or recommendation. Table 3 shows clinical inquiries from DR-TB sites and recommendations from the consilium.

**Table 3:** Clinical inquiries from DR-TB treatment sites and recommendations of the consilium

	**Count (%)**
*Clinical inquiries from DR-TB sites (N = 308)*	
Construction of treatment regimen	119 (38.6)
Management of adverse effect	74 (24.0)
Determination treatment duration	67 (21.8)
Validation of already prescribed regimen	30 (9.7)
Establishment of DR-TB diagnosis	17 (5.5)
Unknown	1 (0.3)
*Recommendations of consilium (N = 471)*	
Construction of initial DR-TB regimen	102 (21.7)
Observation while on current regimen	78 (16.6)
Adherence counselling	66 (14.0)
Dose adjustment	36 (7.6)
Further diagnostic work up	35 (7.4)
Further clinical evaluation by specialist	32 (6.8)
Substitution of single agent in the regimen	30 (6.4)
Change of regimen (substituting 2 or more agents)	25 (5.3)
Adjunct medication	23 (4.9)
Withholding a single agent without substituting it	15 (3.2)
Withholding all DR-TB treatment for a while	13 (2.8)
Other recommendation	16 (3.4)

DR-TB–drug resistant tuberculosis

## Discussion

In this study, we evaluated the characteristics and treatment outcomes of DR-TB cases reviewed by the national DR-TB consilium in Uganda, a TB/HIV high-burdened country. We found that DR-TB facilities mostly inquired about construction of DR-TB regimens and management of side effects. The consilium mostly recommended DR-TB treatment regimens and observation of patients while on a given regimen. Patients reviewed by the consilium were more likely to be from facilities other than MNRH, aged ≥ 35 years, unemployed, defaulted ART, had baseline systolic hypotension, hepatic injury, bilateral hearing loss, psychiatric symptoms, resistance to SLDs and previous exposure to SLDs. Further, reviewed cases were more likely to be prescribed bedaquiline, clofazimine, linezolid and high-dose isoniazid. Treatment success was lower among cases reviewed by the consilium. To our knowledge, this is the first report on cases reviewed by national TB consilia in SSA.

Our results suggest that health workers in peripheral facilities face challenges in constructing regimens for patients with comorbidities and management of adverse drug events. This highlights the need to build the capacity of health workers in these facilities—where most patients were initiated on therapy—to be able to design optimal DR-TB regimens. While there have been efforts to improve health worker skills in DR-TB management in Uganda, there has not been an evaluation of health worker knowledge and practices regarding DR-TB in Uganda [21]. DR-TB knowledge among health workers is consistently low in SSA [24–26]. Similar to our findings, the core clinical questions raised by health workers in Belgium and the United Kingdom (UK) were about the treatment regimen, management of adverse effects, and duration of therapy [16]. In France and the UK, preventive treatment for DR-TB contacts, management of non-tuberculous mycobacterial infection, rifampicin and isoniazid monoresistance, and interpretation of molecular tests were among the clinical questions handled by the consilia, which was not observed in our study [16]. However, from our experience as members of the consilium, these are common clinical queries raised by health workers in Uganda. It is likely that poor documentation of consilium recommendations in the patients’ charts explains why they were not enumerated in our study.

Cases presented to the consilium were more likely to have poor DR-TB prognostic indicators that included advanced age, low socioeconomic status (unemployment as a proxy), drug toxicities (hepatic injury, hearing loss, and psychiatric symptoms), poor ART adherence and more extensive resistance profiles. This is expected because consilia predominantly deal with complex clinical scenarios. It is concerning that patients with severe anaemia were not preferentially reviewed by the consilium yet severe anaemia has been associated with a 91% reduction in the odds of treatment success in this cohort [20]. It is important that the consilium in Uganda prioritises review of patients with anaemia. Similar to our findings, in France, cases were more likely to be presented to the consilium if they had XDR-TB [27]. In the current study, the consilium was more likely to be involved in designing regimens with new DR-TB agents (bedaquiline), repurposed drugs (clofazimine and linezolid) and drugs in short-term treatment regimens (high-dose isoniazid) in tandem with the evolving DR-TB treatment regimens [28]. This demonstrates that the consilium has been acting as a “gatekeeper” for new and repurposed drugs and providing expert advice based on internationally approved guidelines. Other consilia have played a role in the progressive roll out of bedaquiline which was initially provided on compassionate basis before the current recommendation as a core “group A” drug [16, 29].

Treatment outcomes of patients reviewed by the TB consilia elsewhere have not been well described, particularly in SSA. Current reports focus on the performance of the consilia by evaluating the overall treatment outcomes of DR-TB patients in countries with such consilia. D’Ambrosio et al*.*[16] provide an excellent evaluation of the achievements of the different consilia across Europe and Mexico. They report that the treatment success rate in Belarus improved from 37% in 2010 to 54% in 2016 after establishment of the consilia. In Belgium they note that most of the DR-TB patients diagnosed since 2011 benefited from the consilium advice and the country reported a 92.3% treatment success rate for MDR-TB and 100% for XDR-TB. In Mexico, the treatment success rate achieved between 2010, when the consilium was established, to 2017 was 70%. However, it is unclear what the treatment success rate before establishment of the consilium was. In Portugal, the treatment success was observed in 72% of DR-TB patients, 23% of whom were XDR-TB. In Papua New Guinea, the establishment of the tele-based consilium has seen improvement of DR-TB treatment outcomes from 70 to 81%, although it is difficult to attribute these outcomes solely to the establishment of the consilium [29, 30]. The treatment success rate observed among cases reviewed by the consilium in Uganda is commendable considering the several comorbidities, drug adverse effects and high HIV prevalence in the cases. The treatment success rate among the cases is considerably higher than the global DR-TB success rate of 57% [1], although it is below the global DR-TB target of ≥ 75%.

Our study has some limitations. The retrospective nature of the study potentially introduces information bias since we used retrospective data which could have missed certain consilium recommendations. This was minimised by reviewing e-mail correspondence with sites and health care providers who were present in the respective consilia sittings. Notwithstanding, the large sample size of the primary nationwide cohort accorded us the ability to make meaningful comparisons between cases reviewed with those that were not reviewed.

In conclusion, our study showed a commendable treatment success rate among DR-TB cases reviewed by the multidisciplinary TB consilium in Uganda. Our results suggest that health workers in peripheral facilities face challenges in constructing regimens for patients with comorbidities and management of adverse drug events. This highlights the need to build the capacity of health workers in these facilities.

## Data Availability

The datasets used and/or analysed during the current study available from the corresponding author on reasonable request.

## Abbreviations

TB: Tuberculosis
SSA: Sub-Saharan Africa
DR-TB: Drug resistant tuberculosis
MDR-TB: Multi-drug resistant tuberculosis
XDR-TB: Extensively drug resistant tuberculosis
RR: Rifampicin resistance
WHO: World health organisation
HIV: Human immunodeficiency virus
MNRH: Mulago National Referral Hospital
UK: United Kingdom
SLDs: Second-line drugs
ART: Antiretroviral therapy
WHO: World Health organisation
